# Curating and extending data for language comparison in Concepticon and NoRaRe

**DOI:** 10.12688/openreseurope.15380.2

**Published:** 2023-04-28

**Authors:** Annika Tjuka, Robert Forkel, Johann-Mattis List

**Affiliations:** 1 Department of Linguistic and Cultural Evolution, Max Planck Institute for Evolutionary Anthropology, Leipzig, Saxony, 04103, Germany; 2 Chair for Multilingual Computational Linguistics, University of Passau, Passau, Bavaria, 94032, Germany

**Keywords:** Cross-linguistic database, Test-driven data curation, Lexical data, Word properties, Language comparison

## Abstract

Language comparison requires user-friendly tools that facilitate the standardization of linguistic data. We present two resources built on the basis of a standardized cross-linguistic format and show how the data is curated and extended. The first resource, the Concepticon, is a reference catalog for standardized concepts from linguistic research. While curating the Concepticon, we found that a variety of studies in distinct research fields collected information on word properties. However, until recently, no resource existed that contained these data to enable the comparison of the different word properties across languages. This gap was filled by the Database of Norms, Ratings, and Relations (NoRaRe), which is an extension of the Concepticon. Here, we present the major release of both resources - Concepticon Version 3.0 and NoRaRe Version 1.0 - which represents an important step in our data development. We show that extending and adapting the data curation workflow in Concepticon to NoRaRe is useful for the standardization of cross-linguistic datasets. In addition, combining datasets from different research fields enables studies grounded in language comparison. Concepticon and NoRaRe include lexical data for various languages, tools for test-driven data curation, and the possibility for data reuse. The first major release of NoRaRe is also accompanied by a new web application that allows convenient access to the data.

## Plain language summary

There are more than 7,000 spoken and signed languages in the world today. They vary in their vocabulary and structure their lexicon differently to some degree. Standardizing this diversity is a challenge for researchers interested in language comparison. Therefore, we use a set of tools that facilitate the standardization of data so that they become comparable. By implementing these tools, we created a database with a list of words that appear in studies by historical linguists who want to find out how languages are related to each other. Our goal was to create a catalog of standardized words and links to the lists in which they appear. To this end, we reformatted the available data into a simple text file format to make them comparable across languages. This database is called Concepticon. When we added metadata that described various properties of the words to the Concepticon, we noticed that a wealth of additional data existed. These data provide information about when a particular word was acquired, how often it changes its meaning over time, or which words it is associated with. For this reason, we decided to create a separate resource for these types of data: the Database of Norms, Ratings, and Relations (NoRaRe). NoRaRe is an extension of Concepticon and uses a customized workflow to add new data. Here, we introduce the most recent release of Concepticon Version 3.0 and NoRaRe 1.0. We illustrate the content of the two resources, our approach to adding more data, and present a web application that allows for convenient exploration of the data.

## Introduction

The comparison of languages is made possible by standardizing data from various sources. To facilitate this standardization, we need tools to help systematically unify the data and provide them in a FAIR format, i.e., the data need to be *findable*, *accessible*, *interoperable*, and *reusable* ( Wilkinson *et al.*, 2016). Striving for this standard is especially difficult when dealing with linguistic data since languages vary greatly and language scientists choose to structure their data differently. Therefore, it is necessary to create a standardized format that applies to all languages, and at the same time, to provide the tools for effortless standardization. A community-led initiative has recognized this need and developed the Cross-Linguistic Data Formats (CLDF, Forkel *et al.*, 2018) which provide specifications on how to format a given dataset to comply with the FAIR principles ( Wilkinson *et al.*, 2016). The CLDF format specifically targets interoperability and reusability while the storage of the data on Zenodo ( zenodo.org) accounts for findability and accessibility. The advantage of this framework is that cross-linguistic data from diverse languages becomes comparable by converting them into a standardized tabular format and thus, adding new data becomes straightforward. The data curation workflows established through CLDF also allow the curation and extension of data for language comparison. We apply a test-driven data approach, i.e., specific tests are carried out that match the formal requirements of the data automatically with the specifications developed in CLDF.

To compare lexical data across diverse languages, we use the CLDF format to curate resources such as the Concepticon ( List *et al.*, 2016a) and the Database of Norms, Ratings, and Relations (NoRaRe, Tjuka *et al.*, 2022a). The goal of the Concepticon is to equip linguists with a reference catalog of “comparative concepts” ( Haspelmath, 2010) through linking concept lists to standardized concept sets. As the Concepticon continues to develop, data curation workflows have proven useful in adding new data and improving existing data. From the beginning, the Concepticon contained a small number of metadata on word properties including age-of-acquisition ratings (e.g., Kuperman *et al.*, 2012), naming tests (e.g., Ardila, 2007), and links to other databases such as BabelNet ( Navigli & Ponzetto, 2012). However, the data were not continually enriched and a variety of different types of data seemed to be accumulating from different research fields, for example, psychology and natural language processing. We, therefore, decided to construct a new resource, building on the Concepticon but using a customized workflow for the available data. This led to the creation of NoRaRe ( Tjuka *et al.*, 2022a). The goal of NoRaRe is to facilitate exchange between different research fields in order to answer big-picture questions using cross-linguistic comparison. The data in Version 0.2 ( Tjuka *et al.*, 2021) included norms, ratings, and relations from studies in linguistics and psychology offering information on word properties such as word frequencies (e.g., Brysbaert & New, 2009), sensory modality ratings (e.g., Lynott *et al.*, 2020), and similarity estimations (e.g., Hill *et al.*, 2015), among others.

Here, we introduce the major release of Concepticon Version 3.0 ( List *et al.*, 2022a) and NoRaRe Version 1.0 ( Tjuka *et al.*, 2022b). Apart from new data and improvements, the releases include refinements to the accompanying Python packages, the publication of Concepticon and NoRaRe as CLDF datasets and the publication of NoRaRe in a web application built on Cross-Linguistic Linked Data (CLLD, clld.org). The improvements represent an important step in the development of both resources, and we illustrate the data curation workflows implemented in Concepticon Version 3.0 and NoRaRe Version 1.0 below. Due to the scope of the data note, we cannot present the entire background information on both resources (for detailed overviews, see List *et al.*, 2016a; Tjuka *et al.*, 2022a). The interested reader may find additional information on technical details and tutorials on how to use the tools presented here on our blog Computer-Assisted Language Comparison in Practice ( calc.hypotheses.org). Table 1 contains a glossary of relevant terminology.

**Table 1.** Glossary of relevant terms and abbreviations occurring in the text. The table is adapted from Tjuka *et al*. (2022a).

Term	Definition
elicitation gloss	In linguistic fieldwork, an "elicitation gloss" is used to denote a concept in a metalanguage. For example, when English is used as a metalanguage, a researcher would use the elicitation gloss *tree* to elicit the expression for the concept TREE in another language, while a Spanish researcher would use the elicitation gloss *árbol* to elicit the same concept.
concept	We define "concept" as a non-linguistic psychological representation of an object in the world. The standardised concepts in Concepticon are built on the theoretical framework of "comparative concepts" proposed by Haspelmath (2010). These comparative concepts are defined by the researcher as a tool for language comparison. In psychology, there is a longstanding debate about what a “concept” constitutes (cf. Barsalou, 2017; Malt & Majid (2013); Murphy, 2002). In comparison, the discussions in linguistics focus mainly on the term “word meaning” (cf. Bolognesi, 2020; Riemer, 2014). We do not propose a solution for these theoretical discussions.
concept identifier	A “concept identifier” is a unique number that is used to identify a concept set.
concept label	A “concept label” is a unique word or phrase describing a concept set.
concept set	The concept identifier and the concept label together form a “concept set”, for example, 906 TREE. Concept sets in Concepticon are described with additional metadata: a definition, a semantic field, and an ontological category. The concept sets are defined to standardize the concepts which are used for language description and comparison.
concept list	The term "concept list" refers to a compilation of concepts in the form of elicitation glosses. They are used by linguists who want to elicit a concept in a particular language. In contrast to dictionaries, the lists are based on questionnaires and are compiled for language comparison or documentation ( List, 2018). The list is usually a table of elicitation glosses such as *tree*, *you*, *what*, and *bring* which represent the concepts TREE, YOU, WHAT, and BRING.
**Abbreviations**	
CLLD	Cross-Linguistic Linked Data ( clld.org): The overarching project structure under which all of the data are published.
CLDF	Cross-Linguistic Data Formats ( Forkel *et al.*, 2018): The format used to standardize the information provided in linguistic research.
CSVW	CSV on the Web (CSVW): A standard format to represent metadata ( csvw.org).
JSON	JSON (JavaScript Object Notation): A text format which allows for import and export of tabular data ( json.org/json-en.html).
SQL	Structured Query Language (SQL): A programming language to query and manage relational databases.

## Materials and methods

### Concept and word lists

The Concepticon began with the collection of concept lists from studies in historical linguistics using cross-linguistic comparisons to create language family trees. These concept lists include basic vocabulary and cross-linguistically comparable concepts such as HAND, TREE, YOU, or GIVE. Historical linguists have used different versions of these lists to elicit the glosses for the concepts across languages and determine cognates indicating language relatedness. At the time one of the most commonly used lists of concepts was created ( Swadesh, 1955), there was a lack of standardization efforts, so subsequent studies expanded or adapted the original list as they saw fit ( List, 2018). The Concepticon was the first resource to include various concept lists and make them comparable. It enables researchers to find and use the available concept lists for their studies.

On the surface, concept lists look like a list of words. However, words represent concepts in the mind, and in the case of language comparison, there may not be a translational equivalent for a given concept in a language. Similarly, word lists that are used in psychology elicit word properties of concepts to determine whether they are perceived as abstract or concrete, positive or negative, etc. These studies offer additional data on a given word or concept in an individual language and are also integrated into the Concepticon ( List *et al.*, 2016a). While studies in linguistics are comprised of a small set of items, with the rise of large-scale data collection, psychologists publish word lists that include thousands of words. In order to incorporate these data, the Concepticon was extended by establishing the NoRaRe database ( Tjuka *et al.*, 2022a).

The different contents of the concept lists in Concepticon are accounted for by giving them tags which make it more straightforward to search for a particular kind of data ( List, 2018). Table 2 shows the 22 tags we use in the Concepticon ( List *et al.*, 2022a). For example, the tag areal comprises lists that are used to elicit concepts in a particular geographic area such as Vanuatu (e.g., Walworth & Shimelman, 2018). The lists with the tag body parts include studies that elicit body part terms in various languages (e.g., Majid & van Staden, 2015). Lists that are tagged as ranked have a hierarchical order of the words indicating their rates of semantic change (e.g., Pagel *et al.*, 2007). All lists represented in NoRaRe are given at least one of the tags norms, ratings, or relations. Since some lists include different contents, they receive multiple tags. More examples are given in Table 2.

**Table 2.** Tags for the Concepticon concept lists ( List *et al.*, 2022a). Parts of the table are repeated from a blog post (List, 2018). Some lists receive multiple tags, so the total number is higher than the number of lists in the Concepticon.

Tag	Description	Count	Example
acquisition	Concept lists related to studies on language acquisition.	6	( Łuniewska *et al.*, 2019)
annotated	Concept lists that contain additional annotations, for example, on semantic change or colexification degrees. These lists provide extra information beyond the regular content of a concept list.	12	( Urban, 2011)
areal	Concept lists designed to elicit concepts in a specific geographic area.	127	( Walworth & Shimelman, 2018)
basic	Concept lists which are supposed to represent the basic vocabulary.	190	( Sagart *et al.*, 2019)
body parts	Concept lists which concentrate on body parts.	9	( Majid & van Staden, 2015)
colors	A list that is used to elicit color terms.	8	( Haynie & Bowern, 2016)
documentation	Concept lists which serve to document one language or one language family.	5	( Krisadawan, 2000)
hihi	A list of highly reconstructable and highly retentive items (term adopted from McMahon & McMahon, 2005).	3	( McMahon & McMahon, 2005)
lolo	A list of less stable basic items, with low reconstructability and low retentiveness (term adopted from McMahon & McMahon, 2005).	4	( Galucio *et al.*, 2015)
historical	A list which is historically interesting, mostly referring to lists published before the 20 ^th^ century.	13	( Dunn *et al.*, 2017)
naming test	A list designed for a naming test in neurology or psycholinguistics to assess the linguistic capability of children and adults.	5	( Ardila, 2007)
proto-language	A list illustrating the concepts in a proto-language that can be reconstructed with high certainty.	5	( Bodt & List, 2019)
questionnaire	A questionnaire for linguistic fieldwork.	51	( Buck, 1949)
ranked	A list that shows items in a ranked order and has one column reflecting the rank. The ranks are based on, for example, phylogenetic analyses or analysis of borrowing frequencies.	21	( Pagel *et al.*, 2007)
sign language	A list which was designed to investigate sign languages.	3	( Woll *et al.*, 2010)
specific	A list that we consider specific because it is not easily comparable with other lists in the Concepticon. Often the lists include a specific phenomenon studied in linguistics, such as binominal lexemes, pronouns, or semantic groupings.	30	( Pepper, 2020)
stable	A list that is supposed to represent the stable part of a larger list. Usually, the stable part has an unstable counterpart.	8	( Matisoff, 2009)
ultra-stable	A usually very short list of the supposedly most stable concepts.	15	( Pagel *et al.*, 2013)
unstable	A list that is supposed to represent the unstable part of a larger list. Usually has a stable counterpart.	5	( Daniel *et al.*, 2021)
norms	A word list that includes data measured by taking samples from a set, such as word frequency.	6	( Cai & Brysbaert, 2010)
ratings	A word list comprised of ratings of a word by human participants on a scale or other measures, for example, whether a word is positive or negative.	34	( Monnier & Syssau, 2014)
relations	A word list containing various types of semantic relations (e.g., lexical semantic similarity).	11	( Vulić *et al.*, 2020)

### Data curation and reuse

The Concepticon was established in 2015 with its first major release in 2016 including 162 concept lists (Concepticon Version 1.0: List *et al.*, 2016a; List *et al.*, 2016b). Minor releases followed in the subsequent years and the last major release was in 2019 (Concepticon Version 2.0: List *et al.*, 2019). The Concepticon then already contained 240 concept lists with additional links to metadata from studies collecting age-of-acquisition ratings ( Kuperman *et al.*, 2012) and other databases such as BabelNet ( Navigli & Ponzetto, 2012) or OmegaWiki ( OmegaWiki, 2020). At this point, the available metadata were slightly hidden, and we noticed that for words and concepts, there is a whole range of other data on norms, ratings, and relations to be found. Therefore, we decided to launch a satellite project that builds on the established workflows in Concepticon making the available data from linguistics and psychology FAIR ( Wilkinson *et al.*, 2016), especially interoperable and reusable. This is how NoRaRe ( Tjuka *et al.*, 2022a) got started. Since 2019, we are continuously adding new data to Concepticon and NoRaRe. This is the result of our data curation workflows, which are straightforward and can be managed by external and internal collaborators (e.g., student assistants). A list of all contributors to date can be found here: github.com/concepticon/concepticon-data/blob/v3.0.0/CONTRIBUTORS.md. The data curation of Concepticon is organized around the collaboration platform GitHub (concepticon-data repository: github.com/concepticon/concepticon-data). New lists or improvements start out as issues that can be triaged and addressed by the team of editors. If a list is selected for inclusion, the original data is transformed into a tabular format (if necessary), described by a metadata file, and additional metadata is added to the catalog. The concepts or words in the list are then mapped to the Concepticon concept sets (a description of the concept mapping is given below). To facilitate the process for internal and external contributors, we offer documentation under github.com/concepticon/concepticon-data/blob/v3.0.0/CONTRIBUTING.md and blog posts that provide step-by-step tutorials ( Tjuka, 2020a). All improvements and new concept lists are added by creating a pull request (PR) so that the changes can be reviewed by the Concepticon editors. The review process is described in detail in a blog post (see Tjuka, 2021a). The editors are a group of expert linguists who not only check the formal correctness of the contribution but also discuss questions of the content, for example, about concept mappings or additions of new concepts. Integrated into the PR are automated checks of the data that fail if a contribution is flawed. These checks have proven extremely useful since accidental mistakes such as spelling errors or missing concept set identifiers tend to creep into the contribution workflow. This is the advantage of a test-driven data curation workflow and additionally, we have been able to identify mistakes in the original concept list with this process. The NoRaRe database is an extension of the Concepticon and thus, uses similar workflows. Established in 2020 with two minor releases ( Tjuka *et al.*, 2021), the first major release of NoRaRe Version 1.0 includes 113 datasets across 39 languages ^1^ ( Tjuka *et al.*, 2022b). The NoRaRe database is also curated on GitHub (norare-data repository: github.com/concepticon/norare-data). To ensure the same quality of data as in Concepticon, improvements or new lists are added by creating a PR and are reviewed by one of the editors. Currently, the editor team of the NoRaRe database is small but will likely grow in the future. Documentation (see github.com/concepticon/norare-data/tree/v1.0.1) and a step-by-step guide in form of a blog post ( Tjuka, 2021b) are provided as references for internal and external contributors. The data curation is also test-driven in that consistency checks are integrated to identify mistakes easily. In addition, the NoRaRe database offers predefined scripts that allow a quick correlation analysis of the data. The scripts are included in the folder examples here: github.com/concepticon/norare-data/tree/v1.0.1/examples. A blog post described the use of the scripts and how NoRaRe datasets are compared ( Tjuka, 2021c). For example, we compared word frequencies across English, German, and Chinese, and the results showed that word frequencies were similar ( Tjuka, 2020b). Another study compared sensory modality across English, Dutch, and Italian showing subtle differences in each sensory modality ( Tjuka, 2022). Thus, the database can be used to compare word properties and reveal both cross-linguistic similarities and differences.

The result of our data curation efforts is 413 concept lists across 56 languages in Concepticon Version 3.0 ( List *et al.*, 2022a). Concepticon currently has 3,914 concept sets with an average of 231.76 concept sets mapped in a given list. 20,878 unique elicitation glosses are mapped to the Concepticon concept sets. NoRaRe Version 1.0 ( Tjuka *et al.*, 2022b) includes 113 datasets (25 norms, 65 ratings, and 38 relations) across 39 languages and data on 75 properties. Table 3 provides an overview of the descriptive statistics.

**Table 3.** Descriptive statistics of Concepticon 3.0 and NoRaRe 1.0.

	Concepticon 3.0		NoRaRe 1.0
**Lists**	413	**Datasets**	113
**Concepts sets**	3914	**Word properties**	75
**Languages**	56	**Languages**	39
**Language families**	14	**Language families**	8

### Manual and automated mapping

Detailed descriptions of our workflows are provided in the articles introducing Concepticon ( List *et al.*, 2016b) and NoRaRe ( Tjuka *et al.*, 2022a) as well as in the tutorials on our blog ( Tjuka, 2020a; Tresoldi, 2019a; Tresoldi, 2019b). Here, we summarize the basic steps of the manual and automated mapping workflows.

**Figure 1.**
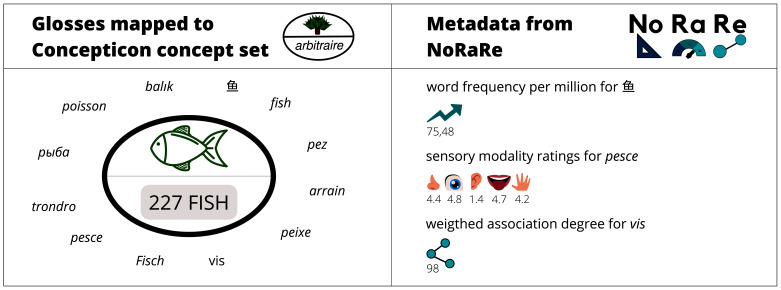
Illustration of the content of the Concepticon ( List *et al.*, 2016a) on the left and NoRaRe ( Tjuka *et al.*, 2022a) on the right.

The first step in the mapping workflow in Concepticon is to generate a mapping to the Concepticon concept sets. The Concepticon concept sets include an identifier, a description, and elicitation glosses linked from each list. Table 4 shows a small subset of the 3,914 Concepticon concept sets. Each concept set has a unique identifier (ID) and a Concepticon gloss. In addition, the semantic field, a description, and the ontological category are provided. An algorithm based on previous mappings generates a list of pre-selected concept sets which are possible for a particular elicitation gloss (examples of elicitation glosses are illustrated in Figure 1). The contributor then checks whether the elicitation gloss represents the proposed Concepticon concept set. For example, depending on the information in the source, the elicitation gloss *bank* needs to be mapped either to the concept set 1284 BANK or 3463 RIVERBANK. It is important to note that we try to map as many elicitation glosses in a list as possible while at the same time, improving the mappings by not mapping an elicitation gloss to a concept set if the meaning cannot be disambiguated. For example, if a list contains the elicitation gloss *smoke* without any further information about whether the verb or noun meaning is intended, we do not map it to one of the concept sets 778 SMOKE (EXHAUST) or 1689 SMOKE (INHALE). Elicitation glosses which are not assigned to a concept set are automatically linked to the concept set 0 NA. The mappings found in the Concepticon are the basis for the automatic mapping workflow used in NoRaRe.

**Table 4.** Examples of Concepticon concept sets across the six ontological categories.

ID	Gloss	Semantic field	Definition	Category
7	GATHER	Spatial relations	To collect or gather (e.g. work, magazines, etc.).	Action */*Process
153	GREY	Sense perception	Having a color between black and white, like ash or stone.	Property
1256	HEAD	The body	The part of the body of an animal or human which contains the brain, mouth, and main sense organs.	Person */*Thing
2451	FOURTEEN	Quantity	The natural number fourteen (14).	Number
2166	PILLOW ( CLASSIFIER)	Quantity	Classifier for pillows and other objects related to the bed.	Classifier
3843	BUT	Miscellaneous function words	A coordinating expression that signals a contrast to what was previously said, e.g. disagreement or a further point to consider.	Other

The automated mapping workflow in NoRaRe is used when it is not feasible to manually check each concept set mapping. Concept and word lists included in the Concepticon are small lists with about 100 to 1,000 items, whereas the basis for lists in NoRaRe can be thousands of words. The datasets in the NoRaRe database are generated by a Python script that automatically downloads the data from the source, i.e., an open repository or web page. Then the raw data are transformed into a tabular format and the words in the list are automatically mapped to the Concepticon concept sets.

For this procedure, the algorithm checks the elicitation glosses that are mapped to a given concept set in Concepticon (e.g., *tree, árbol*) and compares them with the word in a given list (e.g., *tree, forest, wood*). Since the mapping is idempotent the output is unchanged even if the algorithm is run multiple times on the same data. If there is a match, the word is mapped. The result is a reduced list that includes only the words from the original list that were mapped to the Concepticon concept sets. This procedure decreases the file size and avoids consuming an unnecessary amount of data volume. NoRaRe also includes mappings of typed data, for example, entries in Wikidata ( wikidata.org) and BabelNet ( babelnet.org), which is an added benefit compared to the data in Concepticon.

### Python package pyconcepticon

The Python package pyconcepticon ( pypi.org/project/pyconcepticon/3.0.0) supports data curation in Concepticon. To use pyconcepticon, a copy of the Concepticon data must be locally accessible. When pyconcepticon is installed with pip, the integrated commands can be called through the command line. Typing concepticon -h will give a list of the functionalities, for example, create_metadata which automatically creates the metadata JSON file. Guides on how to use the functionalities can be found on our blog ( Tjuka, 2020a; Tresoldi, 2019a; Tresoldi, 2019b).

The pyconcepticon package stores a number of tests that allow for consistency checks of the Concepticon data. Especially, if a new concept list or improvements on existing data are added, the tests that run with the command concepticon test can spot inconsistent mappings, missing files, incorrect numbering of the concept sets, and many more mistakes. To check the consistency of an individual concept list, one can use the command concepticon check. The command concepticon validate inspects the availability of metadata for all concept lists. The pyconcepticon package includes several more commands that also simplify the addition of new lists as well as inspecting the available data.

### Python package pynorare

Similar to the pyconcepticon package, we created a Python package for the curation of the NoRaRe data collection, called pynorare ( pypi.org/project/pynorare/1.0.1). The pynorare package can be installed with pip if a local copy of the NoRaRe data repository is downloaded. The command line is used to access the commands and a list of the functionalities can be retrieved by typing norare -h. Guides on how to use the functionalities can be found on our blog ( Tjuka, 2021b; Tjuka, 2021c). 

The consistency of the data in NoRaRe can be tested with the command norare check which checks the entries in the norare.tsv file ( github.com/concepticon/norare-data/blob/v1.0.1/norare.tsv). The norare.tsv file includes information on all the variables in the NoRaRe datasets. It is important that these entries are consistent and that mistakes are immediately identified. Otherwise, the comparison across datasets would become unfeasible. Individual datasets can be checked for internal consistency by using norare validate. Furthermore, the command norare stats creates a summary statistic across all NoRaRe datasets and calculates the number of concept sets that have at least one link to a NoRaRe dataset as well as the available number of datasets links for each concept set.

## Increasing reusability through Cross-Linguistic Data Formats

Cross-Linguistic Data Formats (CLDF, Forkel *et al.*, 2018) – a format for linguistic data that bundles a set of tables as CSV files with JSON metadata describing the relations between these tables – is used as a distribution format for the Concepticon and NoRaRe data to increase interoperability and reusability ^2^. To curate and package the data in Concepticon and NoRaRe as CLDF datasets, we use the tools in cldfbench ( Forkel & List, 2020). CLDF’s extensibility makes it possible to add custom tables to such a package transparently while keeping the semantics of the fully standardized parts of the data, like the table of languages, intact. But the strict consistency promises that CLDF makes and tools like pycldf ( pypi.org/project/pycldf/1.29.0) enforce also serve as quality control during the data curation workflow to improve reusability. CLDF datasets follow the data model of relational databases, where tables can link to each other using foreign keys. This means that CLDF datasets can automatically be converted to relational databases to facilitate efficient data access. It also means that CLDF datasets follow the recommendations for “tidy” data ( Wickham, 2014; Wilson *et al.*, 2017), in particular by providing unique identifiers for each row of a table. In the context of NoRaRe, another feature of CLDF, or rather the underlying CSVW specification ( pypi.org/project/csvw/3.1.3), also helps with quality control. CSVW metadata can specify datatypes for data in CSV files, and thus augment the “raw” text data with well-defined conversions to typed data. For NoRaRe, this turns out to be particularly important, because NoRaRe variables provide many different types of data, ranging from continuous numbers from a limited range to categorical variables, with string values from a controlled vocabulary. The corresponding CSVW datatype descriptions then serve as documentation of valid assumptions for data reuse, but also as specifications for data consistency checks, which are built-in to CLDF validation.

### CLDF datasets

With the major release of Concepticon Version 3.0 ( List *et al.*, 2022a) and NoRaRe Version 1.0 ( Tjuka *et al.*, 2022b), both resources were also made available as CLDF datasets ( List *et al.*, 2022b; Tjuka *et al.*, 2022c). While the raw data is updated and improved consistently in the respective GitHub repositories, the CLDF datasets allow for the reuse and exploration of the data in a different way. The data in the CLDF datasets are represented in tabular format and the corresponding metadata in JSON format. The bundling of the data makes it possible to represent relations between the tables. The data model is available here: github.com/concepticon/concepticon-cldf/blob/v3.0.0/cldf/README.md. By releasing the data in Concepticon and NoRaRe as CLDF datasets, tools such as csvkit (pypi.org/project/csvkit/1.1.1) can be used to process and analyse the data conveniently. Examples of how to use the Concepticon data from the CLDF dataset can be found under ( pypi.org/project/csvkit/1.0.7) allow the processing and in-depth study of the available data in Concepticon and NoRaRe. Examples of how to use the Concepticon data from the CLDF dataset can be found under github.com/concepticon/concepticon-cldf/tree/v3.0.0/doc and for NoRaRe under github.com/concepticon/norare-cldf/tree/v1.0.0.

An advantage of the CLDF datasets is that CLDF includes the information to load the data from Concepticon or NoRaRe into a relational database and perform various queries. The Python package pycldf ( pypi.org/project/pycldf/1.29.0) can convert any CLDF dataset into an SQLite database that allows queries with SQL. This process replicates the construction of the web applications described below. For Concepticon, queries could include listing the Concepticon concept sets in a given concept list or showing concept set relations such as plotting all the *narrower* concept sets connected to 1262 BROTHER, i.e., 559 BROTHER ( OF MAN), 560 BROTHER ( OF WOMAN), 1759 OLDER BROTHER, 1760 YOUNGER BROTHER, etc. The relations *broader* versus *narrower* are used to indicate concept sets that refer to specific parts of a more general concept. For example, we established the broader concept set 3626 KNOW to relate it to the narrower concept sets 1410 KNOW (SOMETHING) and 2248 KNOW (SOMEONE) because we found that several concept lists did not provide a clear distinction between the two concepts. It is important to note that the Concepticon does not provide a full ontology but rather that the relations are established bottom-up and mainly serve as an exploration of the data instead of providing a basis for inferences. For NoRaRe, one could query word frequencies for words expressing the same concept. Another possibility would be to compute correlations by assembling data from different datasets, for example, arousal ratings from different languages. The use of the Python packages pandas ( pypi.org/project/pandas/1.5.1) and seaborn ( pypi.org/project/seaborn/0.12.1) allows the creation of a dot plot for the correlation. Note that variables may have multiple values assigned to the same concept set because different words were mapped to the same concept set. Although these cases are rare, researchers need to inspect the mapped words before deciding whether or not they want to include them in a correlation study.

### CLLD web applications

**Figure 2.**
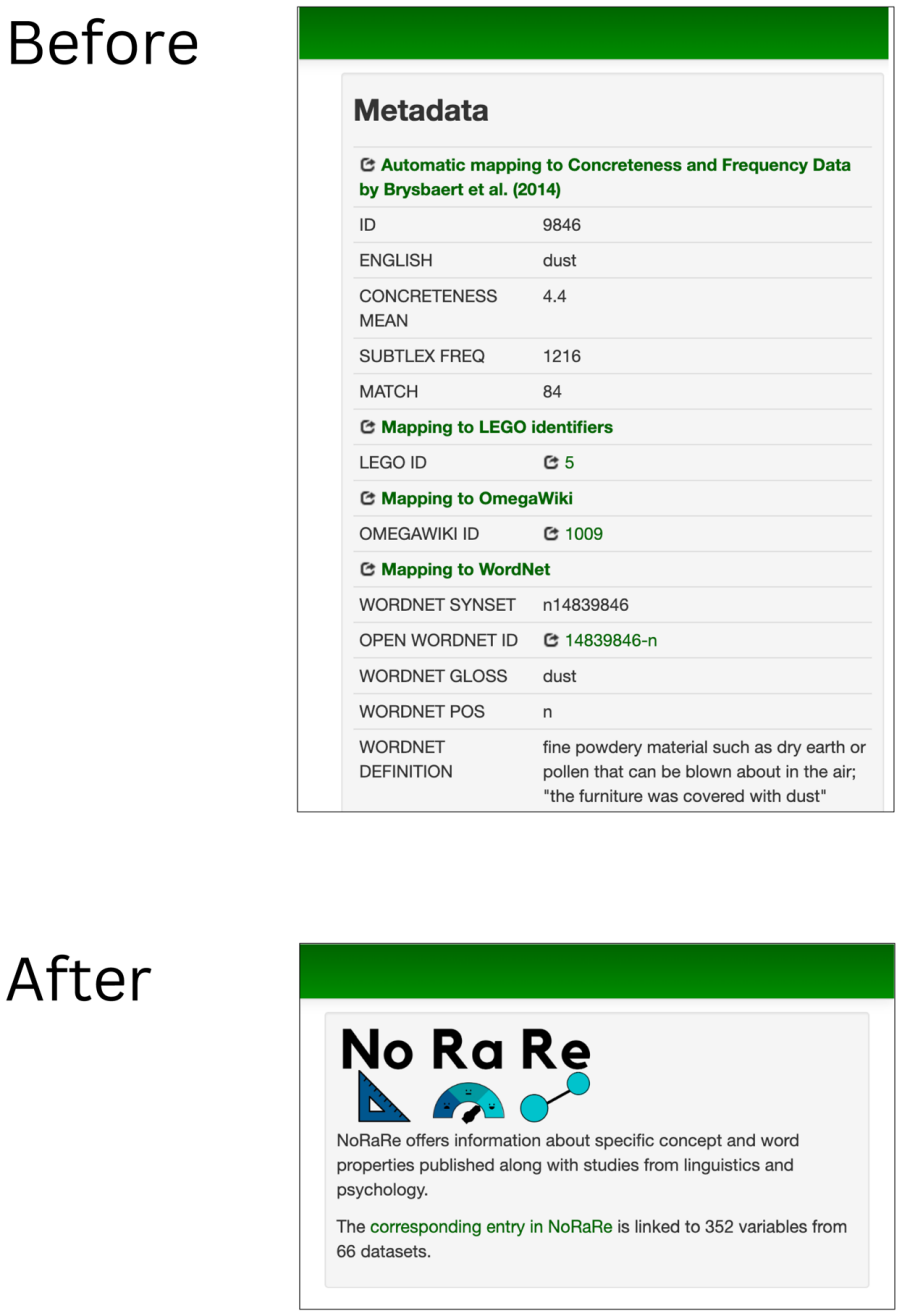
Replacement of the metadata box with a link to NoRaRe for each concept set.

The CLDF datasets described above are the input for the clld applications developed in the Cross-Linguistic Linked Data (CLLD) project ( clld.org and documentation under clld.readthedocs.io/en/latest). The CLLD project allows the curation and development of lexical and grammatical databases. The clld toolkit ( pypi.org/project/clld/10.0.0) is a Python package that integrates functions for building and maintaining CLLD web applications. These web applications (short: *web apps*) can be conveniently accessed via a web browser. They are also a good way to check the consistency of data and are a form of data reuse. The CLDF datasets of Concepticon and NoRaRe include all the information to create clld web apps for each data collection. Each new data release is accompanied by an update of the web applications. The clld web application for Concepticon was already introduced with the first release of Concepticon Version 1.0 ( List **et al.*,* 2016b) in 2016. The major release of Concepticon 3.0 ( List **et al.*,* 2022a) and NoRaRe 1.0 ( Tjuka **et al.*,* 2022b) brings a number of updates that affect the presentation of data. In the previous version of the Concepticon web application, different kinds of metadata on word frequency, concreteness ratings, links to WordNet ( Miller *et al.*, 1990), etc. were represented in a box beside the elicitation glosses linked to a given concept set. The most significant change for the Concepticon web app ( concepticon.clld.org) apart from the data update is the integration of a link to the NoRaRe data and a summary statistic indicating the number of links to variables and datasets for each concept set that replace the metadata box (see Figure 2).

**Figure 3.**
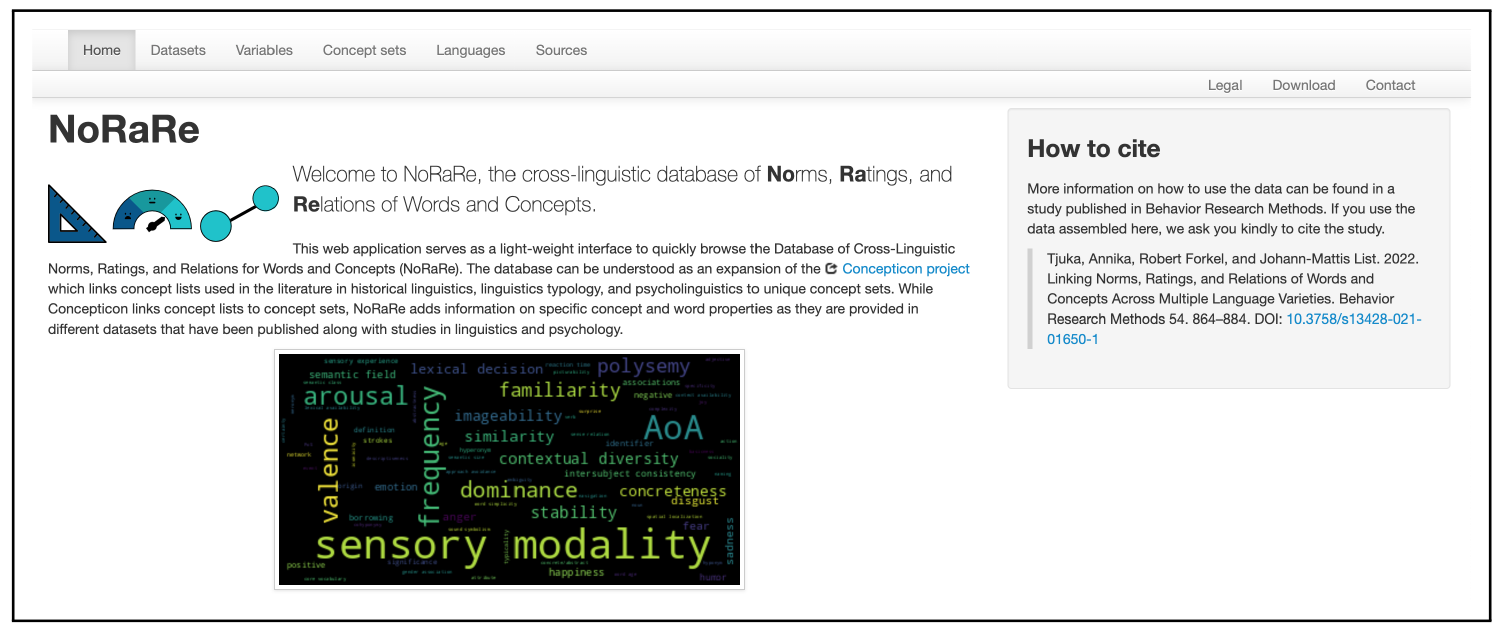
Word cloud illustrating the tags used for each variable in NoRaRe ( norare.clld.org).

**Figure 4.**
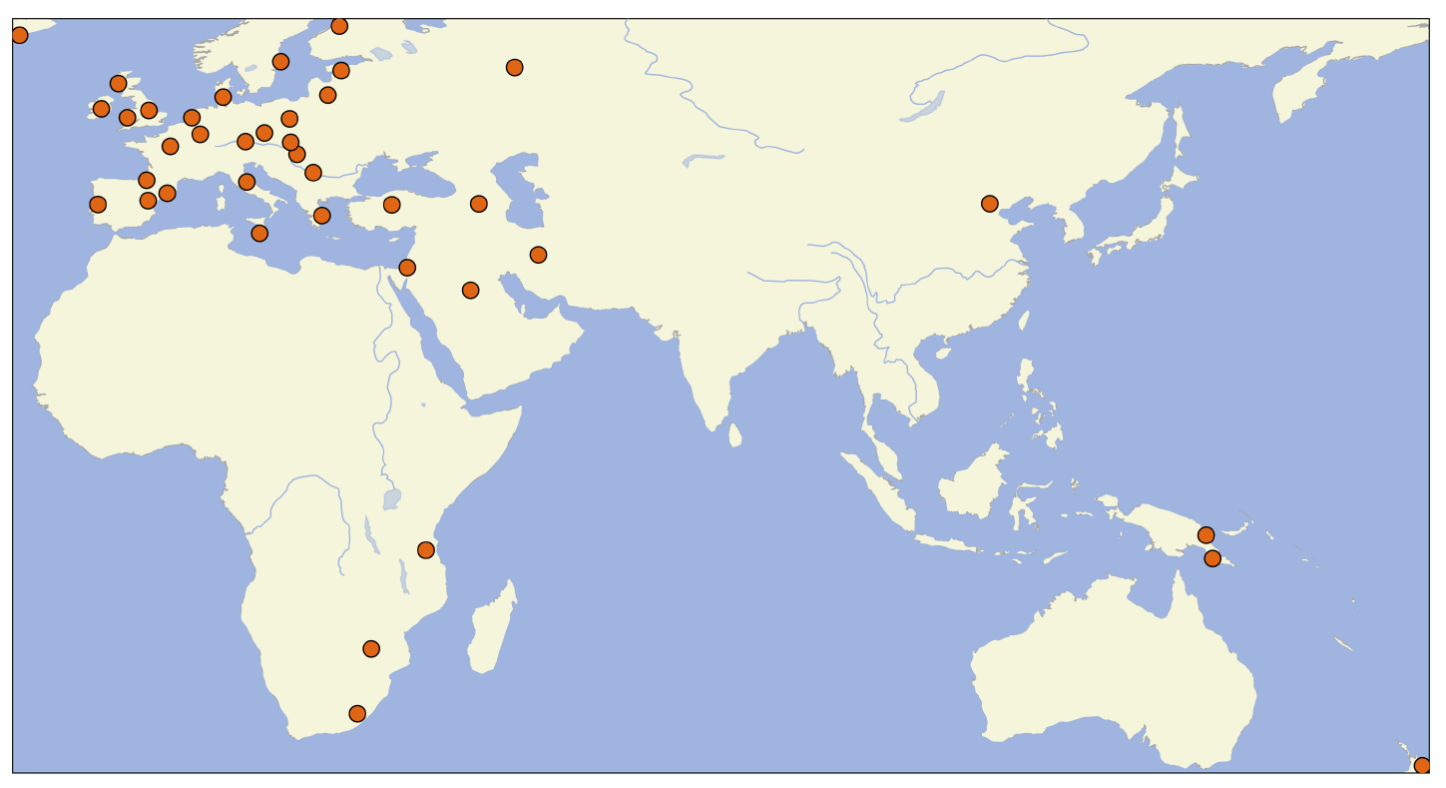
Distribution of languages in the NoRaRe datasets.

The clld web app for NoRaRe ( norare.clld.org) was introduced for the first time with the major release of NoRaRe Version 1.0 ( Tjuka *et al.*, 2022b). The NoRaRe web application has a similar structure to the Concepticon web application while at the same time, the features of NoRaRe including a list of all datasets and the variables for each concept set are highlighted. The word cloud on the front page is automatically generated based on the tags used for each variable in NoRaRe (see Figure 3). The font size represents the frequency of the individual tags across all datasets in NoRaRe. Most NoRaRe datasets include multiple variables which become apparent by clicking on the link for a given dataset. The web application also shows which glosses are mapped to a Concepticon concept set. A map illustrates the distribution of languages associated with each value (see Figure 4). Since many datasets containing norms, ratings, and relations come from psychological studies, the bias toward Central European languages is obvious. However, once cross-linguistic data from linguistics is added, the distribution of languages extends to areas such as Africa and New Guinea.

## Conclusion

The present article introduced the major release of the cross-linguistic databases Concepticon Version 3.0 ( List *et al.*, 2022a) and NoRaRe Version 1.0 ( Tjuka *et al.*, 2022b). We discussed the contents of both resources and their underlying data curation workflows. The Concepticon includes standardized concept sets that allow comparison across many languages. NoRaRe offers data on norms, ratings, and relations for words and concepts and is an extension of the Concepticon. With the major release, new data were added, the data was published in CLDF format and for NoRaRe, a web application was created.

Our data curation workflows have proven applicable for the advancement of data for language comparison and we envision that other researchers use the two resources for their studies. The availability of the data as CLDF datasets and the Python packages make it possible to explore and test the data more conveniently. Web applications for Concepticon and NoRaRe offer an additional overview of the available data. At this point, Concepticon includes 413 concept lists with 56 languages and 3,914 concept sets. NoRaRe contains 113 datasets with 75 word properties across 39 languages. We intend to further expand these data collections in the future.

## Ethical approval and consent

Ethical approval and consent were not required.

## Data Availability

The Concepticon is curated on GitHub ( github.com/concepticon/concepticon-data/tree/v3.0.0) and archived with Zenodo under CC-BY license ( doi.org/10.5281/zenodo.7296458) ( List *et al.*, 2022a). The CLDF dataset for Concepticon is published on GitHub ( github.com/concepticon/concepticon-cldf/tree/v3.0.0) and Zenodo under CC-BY license ( doi.org/10.5281/zenodo.7298023) ( List *et al.*, 2022b). NoRaRe is also curated on GitHub ( github.com/concepticon/norare-data/tree/v1.0.1) and archived with Zenodo under CC-BY license ( doi.org/10.5281/zenodo.7298060) ( Tjuka *et al.*, 2022b). The CLDF dataset for NoRaRe is published on GitHub ( github.com/concepticon/norare-cldf/tree/v1.0.0) and Zenodo under CC-BY license ( doi.org/10.5281/zenodo.7312927) ( Tjuka *et al.*, 2022c). Data are available under the terms of the Creative Commons Attribution 4.0 International license (CC-BY 4.0). The Python packages used for the data curation workflows can be found on PyPi under Apache license: pyconcepticon ( pypi.org/project/pyconcepticon/3.0.0) and pynorare ( pypi.org/project/pynorare/1.0.1). For convenient access, we offer a CLLD web app for Concepticon ( concepticon.clld.org) and NoRaRe ( norare.clld.org).

## Footnotes

^1^ Note that we counted Cantonese and Mandarin Chinese as two separate languages when introducing NoRaRe ( Tjuka *et al.*, 2022a). The data are now grouped under Chinese because some studies do not clearly distinguish the two dialects.

^2^ We refer readers interested in the major design principles behind CLDF and the contrasts with other standardization efforts to the original publication introducing CLDF by Forkel *et al.* (2018).
