# Supplementary material for: Curating and extending data for language comparison in Concepticon and NoRaRe
Source: Open Res Eur. 2023 May 24;2:141. Originally published 2022 Dec 16. [Version 3] doi: 10.12688/openreseurope.15380.3 (PMC10446050; doi:10.12688/openreseurope.15380.3)
Supplement: Peer review history

# Peer review: Reviewer response for version 3

**DOI:** 10.21956/openreseurope.17368.r32031

**Published:** 2023-05-25

**Authors:** Gemma Boleda

**Affiliations:** 1 Department of Translation and Language Sciences, ICREA, Catalan Institution for Research and Advanced Studies, Universitat Pompeu Fabra, Barcelona, Spain

No further comments.

Are sufficient details of methods and materials provided to allow replication by others?

No

Is the rationale for creating the dataset(s) clearly described?

Yes

Are the datasets clearly presented in a useable and accessible format?

Yes

Are the protocols appropriate and is the work technically sound?

Partly

Reviewer Expertise:

Semantics, lexicon, linguistics, computational linguistics, cognitive science

I confirm that I have read this submission and believe that I have an appropriate level of expertise to confirm that it is of an acceptable scientific standard.

# Peer review: Reviewer response for version 2

**DOI:** 10.21956/openreseurope.17268.r31333

**Published:** 2023-05-15

**Authors:** Gemma Boleda

**Affiliations:** 1 Department of Translation and Language Sciences, ICREA, Catalan Institution for Research and Advanced Studies, Universitat Pompeu Fabra, Barcelona, Spain

I congratulate the authors for the revision. There are only two minor remaining changes I would like to see in the article:

- add this clarification that you gave in the response to the body of the article: "The ontological categories are based on the categories used by the World Loanword Database (Haspelmath and Tadmor 2009). However, they were modified regarding the concrete terms used, in order to make clear that we do not talk about concrete parts of speech since we are dealing with concepts and not with words (see List et al. 2016: 2394)."
- It is still not clear what "typed data" are; can you provide a small explanation or paraphrase? (This concerns "NoRaRe also includes mappings of typed data, for example, entries in Wikidata ( www.wikidata.org) and BabelNet ( www.babelnet.org), which is an added benefit compared to the data in Concepticon.")

Are sufficient details of methods and materials provided to allow replication by others?

No

Is the rationale for creating the dataset(s) clearly described?

Yes

Are the datasets clearly presented in a useable and accessible format?

Yes

Are the protocols appropriate and is the work technically sound?

Partly

Reviewer Expertise:

Semantics, lexicon, linguistics, computational linguistics, cognitive science

I confirm that I have read this submission and believe that I have an appropriate level of expertise to confirm that it is of an acceptable scientific standard.

# Response

**Published:** 2023-05-16

**Authors:** Annika Tjuka

**Affiliations:** Department of Linguistic and Cultural Evolution, Max Planck Institute for Evolutionary Anthropology, Leipzig, Germany

We thank the reviewer for the positive assessment of our revised manuscript. We implemented the two suggestions and uploaded a final version of the manuscript.

# Peer review: Reviewer response for version 2

**DOI:** 10.21956/openreseurope.17268.r31332

**Published:** 2023-05-02

**Authors:** Thanasis Georgakopoulos

**Affiliations:** 1 School of English, Department of Theoretical and Applied Linguistics, Aristotle University of Thessaloniki, Thessaloniki, Greece

No further comments to make.

Are sufficient details of methods and materials provided to allow replication by others?

Yes

Is the rationale for creating the dataset(s) clearly described?

Yes

Are the datasets clearly presented in a useable and accessible format?

Yes

Are the protocols appropriate and is the work technically sound?

Yes

Reviewer Expertise:

Historical Linguistics; Lexical Semantics; Lexical Typology

I confirm that I have read this submission and believe that I have an appropriate level of expertise to confirm that it is of an acceptable scientific standard.

# Peer review: Reviewer response for version 1

**DOI:** 10.21956/openreseurope.16625.r30509

**Published:** 2023-02-22

**Authors:** Thanasis Georgakopoulos

**Affiliations:** 1 School of English, Department of Theoretical and Applied Linguistics, Aristotle University of Thessaloniki, Thessaloniki, Greece

**1. General overview and assessment**

 The publication of this data note marks the release of two important resources, namely Concepticon (Version 3.0) and NoRaRe (Version 1.0), which come with new data and various improvements over their predecessors. The joint presentation of Concepticon and NoRaRe is noteworthy as they share several common features but also exhibit certain differences. Specifically, Concepticon consists of data from over 400 concept lists, which are collections of concepts put together by different scholars at various times. These lists contain concept labels that refer to the same concept, which are referred to as concept sets. The current version of Concepticon contains more than 3,900 such concept sets, which allow comparison between multiple languages. NoRaRe is considered an extension of Concepticon with a wider scope, designed to foster the dialogue between different disciplines, mainly psychology and linguistics. It consists of three different types of data, namely norms (No), ratings (Ra), and relations (Re), which can be used to compare different concept/word properties across languages.

 The protocols used for the creation of the resources are deemed appropriate, and the work carried out by the editors is technically sound. Both resources tackle the difficult task of standardizing cross-linguistic datasets, which is widely recognized as a challenging undertaking. Curators employ a combination of manual, automated, and semi-automated workflows to maintain and add new data. Both Concepticon and NoRaRe are curated using the GitHub platform and are archived under the CC-BY license on Zenodo. In addition, they adhere to the Cross-Linguistic Data Formats Initiative (CLDF; see Forkel et al., 2018; https://cldf.clld.org), which aims at representing lexical data in straightforward tabular formats. As a result, the datasets are presented in a useable and accessible format. Overall, all datasets comply with the FAIR principles, according to which the data should be *findable*, *accessible, interoperable*, and *reusable* (Wilkinson et al., 2016). Finally, these CLDF datasets are also available on GitHub and serve as input for the CLLD web applications for Concepticon (concepticon.clld.org) and NoRaRe (norare.clld.org).

 The authors provide adequate information about their methods and materials, which enables researchers to reproduce the results of studies that utilize the data provided by these resources. Additionally, this information allows for the addition of new data to the resources by interested researchers. It is worth mentioning that several technical issues are discussed in blog posts authored by the editors, which may further encourage the use of these resources (and which can be seen as complementary to this data note).

 In light of the growing need for standardized cross-linguistic datasets and their importance in enabling reproducible research in linguistics, the newer versions of Concepticon and NoRaRe are a timely and valuable contribution. Therefore, I recommend accepting this data note (with minor changes; see my comments below).

**2. Specific comments (on pdf pages)**

- p. 5: *The authors write that “* *Concepticon currently has 3,914 concept sets* *with an average of 231.76 concept sets mapped in a given list.* *”*
  - It would be informative to learn which lists have the highest and lowest coverage of concept sets. A table featuring the top 5 and bottom 5 lists would be sufficient.

- p. 5: *In the paper, the term ‘elicitation gloss’ is used.*
  - The authors can provide further clarification on the term (and the rationale behind its use). My understanding is that the term "elicitation gloss” is equivalent to "word" in NoRaRe, whereas in Concepticon, it is equivalent to "concept." This difference could potentially be confusing for researchers who intend to use both resources. As was done in Tjuka et al. (2022a), it would be helpful if the authors could elaborate on the distinction between concepts and word forms and the reasons why one could use them interchangeably.

- p. 6: *The authors write that “* *It is important to note that we try to* *map as many elicitation glosses in a list as possible while at the same time, improving the mappings by not mapping an* *elicitation gloss to a concept set if the meaning cannot be* *disambiguated.* *”*
  - Providing an example where the described limitation made it impossible to create a mapping would be helpful for the reader.

- p. 7: *The authors write that “the fact that CLDF datasets can be converted to relation databases turning relations between tables into foreign key constraints implies that valid CLDF datasets will have unique identifiers for each row of a table*.
  - Could you offer additional clarification on the term "foreign key constraints" as used in this context? As far as I understand, it pertains to a set of rules that ensure that the values in one table correspond to the values in another table. Nevertheless, it may be beneficial to provide further explanation here.

- p. 7: The URL for the Python package pyconcepticon ( pypi.org/project/pyconcepticon/3.0.0) leads to the Concepticon collection, whereas the URL for the Python package pynorare ( pypi.org/project/pynorare/1.0.1) does not provide direct access to the NoRaRe collection.

- p. 7:  The authors make reference to csvkit ( pypi.org/project/csvkit/1.0.7), a tool that enables the processing and detailed analysis of the data in Concepticon and NoRaRe. It is worth noting that a newer version (1.1.0) of this tool is available.

- P 7: For readers who are not acquainted with the Concepticon, the term *"narrow concept set"* may be unfamiliar. Could you please provide further explanation of the distinction between broad and narrow concepts (even though the content becomes obvious with the examples that follow)?

- p. 7: *The authors write* *: “1,262* *BROTHER, i.e., 559 BROTHER (OF MAN), 560 BROTHER* *(OF WOMAN), 1,759 OLDER BROTHER, 1760 YOUNGER”*
  - Please remove the commas in the numbers. The correct text should read as follows: "1262 BROTHER, i.e., 559 BROTHER (OF MAN), 560 BROTHER (OF WOMAN), 1759 OLDER BROTHER, 1760 YOUNGER BROTHER."

- p. 8:  The authors may refer to the newer version of the clld toolkit (clld 10.0.0)

Are sufficient details of methods and materials provided to allow replication by others?

Yes

Is the rationale for creating the dataset(s) clearly described?

Yes

Are the datasets clearly presented in a useable and accessible format?

Yes

Are the protocols appropriate and is the work technically sound?

Yes

Reviewer Expertise:

Historical Linguistics; Lexical Semantics; Lexical Typology

I confirm that I have read this submission and believe that I have an appropriate level of expertise to confirm that it is of an acceptable scientific standard.

## References

[1] : Cross-Linguistic Data Formats, advancing data sharing and re-use in comparative linguistics. *Scientific Data* .2018;5(1) : 10.1038/sdata.2018.205 10.1038/sdata.2018.205 PMC619074230325347

[2] : Linking norms, ratings, and relations of words and concepts across multiple language varieties. *Behavior Research Methods* .2021;54(2) : 10.3758/s13428-021-01650-1 864-884 10.3758/s13428-021-01650-1 PMC904630734357536

[3] : The FAIR Guiding Principles for scientific data management and stewardship. *Scientific Data* .2016;3(1) : 10.1038/sdata.2016.18 10.1038/sdata.2016.18 PMC479217526978244

[4] : Concepticon 2.6.0. Max Planck Institute for Evolutionary Anthropology.2022; Reference source

# Response

**Published:** 2023-02-27

**Authors:** Annika Tjuka

**Affiliations:** Department of Linguistic and Cultural Evolution, Max Planck Institute for Evolutionary Anthropology, Leipzig, Germany

We thank the reviewer for this detailed assessment of our manuscript. We will now start preparing a new version of the manuscript, addressing the points raised in the two reviews.

# Response

**Published:** 2023-04-21

**Authors:** Annika Tjuka

**Affiliations:** Department of Linguistic and Cultural Evolution, Max Planck Institute for Evolutionary Anthropology, Leipzig, Germany

We would like to thank the reviewer for the extensive and detailed summary of our data note. It shows that the manuscript was carefully evaluated in light of the larger context of our study. The point regarding our blog encouraged us to include a reference to it in the introduction. We implemented the suggested changes and respond to the reviewer’s comments below (order: reviewer comment, our response, implemented change).

- p. 5: The authors write that “Concepticon currently has 3,914 concept sets with an average of 231.76 concept sets mapped in a given list.” It would be informative to learn which lists have the highest and lowest coverage of concept sets. A table featuring the top 5 and bottom 5 lists would be sufficient.

We thank the reviewer for this suggestion. In light of the comments from reviewer 1, who suggested a table with a different set of descriptive statistics, we decided to include only one additional table (Table 3). However, we would like to refer the reviewer to the Concepticon GitHub repository, where additional statistics can be found, which are updated with each release. Furthermore, the "Representation" tab on the Concepticon website shows how many concept lists are mapped to a specific concept set. The list can be sorted to show the highest and lowest number of coverage. The relevant links are listed below.

- https://github.com/concepticon/concepticon-data/tree/v3.0.0/concepticondat
- https://concepticon.clld.org/parameters 

Table 3:   Concepticon 3.0.   NoRaRe 1.0. Lists 413 Datasets 113 Concepts sets 3914 Word properties 75 Languages 56 Languages 39 Language families 14 Language families 8  

- p. 5: In the paper, the term ‘elicitation gloss’ is used. The authors can provide further clarification on the term (and the rationale behind its use). My understanding is that the term "elicitation gloss” is equivalent to "word" in NoRaRe, whereas in Concepticon, it is equivalent to "concept." This difference could potentially be confusing for researchers who intend to use both resources. As was done in Tjuka et al. (2022a), it would be helpful if the authors could elaborate on the distinction between concepts and word forms and the reasons why one could use them interchangeably.

We thank the reviewer for the suggestion. To clarify the use of the terms, we included a glossary at the beginning of the manuscript (Table 1). Table 1: Term Definition elicitation gloss In linguistic fieldwork, an "elicitation gloss" is used to denote a concept in a metalanguage. For example, when English is used as a metalanguage, a researcher would use the elicitation gloss tree to elicit the expression for the concept TREE in another language, while a Spanish researcher would use the elicitation gloss árbol to elicit the same concept.  

- p. 6: The authors write that “It is important to note that we try to map as many elicitation glosses in a list as possible while at the same time, improving the mappings by not mapping an elicitation gloss to a concept set if the meaning cannot be disambiguated.”
  Providing an example where the described limitation made it impossible to create a mapping would be helpful for the reader.

We integrated the suggestion of the reviewer by adding the following sentence. "For example, if a list contains the gloss *smoke* without any further information about whether the verb or noun meaning is intended, we do not map it to one of the concept sets 778 SMOKE (EXHAUST) or 1689 SMOKE (INHALE)."  

- p. 7: The authors write that “the fact that CLDF datasets can be converted to relation databases turning relations between tables into foreign key constraints implies that valid CLDF datasets will have unique identifiers for each row of a table.
  Could you offer additional clarification on the term "foreign key constraints" as used in this context? As far as I understand, it pertains to a set of rules that ensure that the values in one table correspond to the values in another table. Nevertheless, it may be beneficial to provide further explanation here.

Reviewer 1 had similar concerns about the sentence so we paraphrased it to make the terminology clearer. "CLDF datasets follow the data model of relational databases, where tables can link to each other using foreign keys. This means that CLDF datasets can automatically be converted to relational databases to facilitate efficient data access. It also means that CLDF datasets follow the recommendations for “tidy” data (Wickham 2014; Wilson et al., 2017), in particular by providing unique identifiers for each row of a table."  

- p. 7: The URL for the Python package pyconcepticon (pypi.org/project/pyconcepticon/3.0.0) leads to the Concepticon collection, whereas the URL for the Python package pynorare (pypi.org/project/pynorare/1.0.1) does not provide direct access to the NoRaRe collection.

We thank the reviewer for the thorough check of our link collection. We decided to reference a link to the PyPI page rather than GitHub for the Python packages. Now that data access works via CLDF, pynorare and pyconcepticon are only implementation details. We have stopped archiving releases on Zenodo for Python packages since PyPI is the authoritative archive. That is why we no longer make releases on GitHub (which only served to move the Python package to Zenodo).  

- p. 7:  The authors make reference to csvkit (pypi.org/project/csvkit/1.0.7), a tool that enables the processing and detailed analysis of the data in Concepticon and NoRaRe. It is worth noting that a newer version (1.1.0) of this tool is available.

We updated the link to the newest version of csvkit as suggested by the reviewer. "Tools such as csvkit ( pypi.org/project/csvkit/1.1.1) allow the processing and in-depth study of the available data in Concepticon and NoRaRe."  

- P 7: For readers who are not acquainted with the Concepticon, the term "narrow concept set" may be unfamiliar. Could you please provide further explanation of the distinction between broad and narrow concepts (even though the content becomes obvious with the examples that follow)?

We added further information on the broader/narrower concept distinction. "The relations broader versus narrower are used to indicate concept sets that refer to specific parts of a more general concept. For example, we established the broader concept set 3626 KNOW to relate it to the narrower concept sets 1410 KNOW (SOMETHING) and 2248 KNOW (SOMEONE) because we found that several concept lists did not provide a clear distinction between the two concepts. It is important to note that the Concepticon does not provide a full ontology but rather that the relations are established bottom-up and mainly serve as an exploration of the data instead of providing a basis for inferences."  

- p. 7: The authors write: “1,262 BROTHER, i.e., 559 BROTHER (OF MAN), 560 BROTHER (OF WOMAN), 1,759 OLDER BROTHER, 1760 YOUNGER” Please remove the commas in the numbers. The correct text should read as follows: "1262 BROTHER, i.e., 559 BROTHER (OF MAN), 560 BROTHER (OF WOMAN), 1759 OLDER BROTHER, 1760 YOUNGER BROTHER."

The commas were included in the typesetting of the manuscript. We thank the reviewer for noticing them. We deleted all additional commas in the identifiers of concept sets in the revision. "For Concepticon, queries could include listing the Concepticon concept sets in a given concept list or showing concept set relations such as plotting all the narrower concept sets connected to 1262 BROTHER, i.e., 559 BROTHER (OF MAN), 560 BROTHER (OF WOMAN), 1759 OLDER BROTHER, 1760 YOUNGER BROTHER, etc."  

- p. 8:  The authors may refer to the newer version of the clld toolkit (clld 10.0.0)

We updated the link to the newest version of clld toolkit as suggested by the reviewer. "The clld toolkit (pypi.org/project/clld/10.0.0) is a Python package that integrates functions for building and maintaining CLLD web applications."

# Peer review: Reviewer response for version 1

**DOI:** 10.21956/openreseurope.16625.r30510

**Published:** 2023-02-20

**Authors:** Gemma Boleda

**Affiliations:** 1 Department of Translation and Language Sciences, ICREA, Catalan Institution for Research and Advanced Studies, Universitat Pompeu Fabra, Barcelona, Spain

SUMMARY

 This article presents major releases of two large-scale datasets (and accompanying code and web apps) for research in cross-linguistic phenomena related to the lexicon: Concepticon Version 3.0 and NoRaRe Version 1.0. Concepticon provides a standardized set of concepts that are linked to words/concepts in 413 pre-existing datasets; and NoRaRe links the corresponding words (and their concepts from Concepticon) to word properties available in 113 pre-existing datasets.

 Both datasets represent huge compilation and standardization efforts that are very useful for the community and allow for much larger-scale research than was possible before. Furthermore, the way the authors have made the data public follows FAIR principles: the data are easily findable, accessible, interoperable, and reusable. I particularly like it that they have built web apps that make browsing the datasets easy. Another great aspect is that the authors have worked towards a workflow that allows external people to contribute to the datasets. This is an aspect that merits more discussion in the paper.

 While the work itself seems to be excellent (as far as I can tell; see below for missing information, which I need to be able to properly evaluate it) and is certainly relevant for the research community, the reporting in the article needs quite a bit of work. In particular, it should provide more information and less technical detail, as well as structure the information better, as follows.

 ASPECT TO IMPROVE #1: MORE INFORMATION

 The article should be self-contained and provide sufficient information about the dataset for the reader to be able to understand both how it was constructed and the information it contains. The following needs to be addressed:

- Please include a table with relevant descriptive statistics of the datasets in their 3.0 and 1.0 releases (minimally n. of datasets, concepts, words, languages, language families).
- The main notions around which the data are organized should be more clearly explained, with examples. The notion of "concept set" was particularly hard for me to understand; I had to check the websites, and even there it took some digging: The example on the homepage of the CLLD web app ( https://concepticon.clld.org/) talks about the concept set SIBLING, so it looks like the other concepts in the network shown (OLDER SIBLING, YOUNGER BROTHER, ...) are members of this set. However, if one queries the interface, OLDER SIBLING and YOUNGER BROTHER are themselves concept sets. It turns out that "concept set" does not refer to sets of concepts in Concepticon, but to sets of concepts defined in the primary datasets. Please explain and illustrate how you use the following terms: "concept", "concept set", "concept label", "concept list". Also explain how these terms relate to words as commonly defined by linguists, and what the place of words is in the Concepticon (including improving the claim that "concept lists look like a list of words. However, words represent concepts in the mind" -- check e.g. G. Murphy's chapter on word meaning in The Big Book of Concepts).
- Also provide information about the ontological categories: how was the set of categories defined, and how were they assigned to specific concepts?
- The mapping between the primary datasets and Concepticon includes an automatic step: "the algorithm checks the elicitation glosses that are mapped to a given concept set in Concepticon and compares them with the word in a given list. (...) If there is a match, the word is mapped." Please explain how the checking and comparing is made, and what the criteria are to decide whether there is a match, at an adequate level of abstraction for the present report. Also, explain "elicitation glosses" -- I thought they were definitions but Figure 1 suggests they are words. Maybe it depends on the dataset?
- Also please provide information on how many datapoints from the primary sources are left unlinked at present (after the manual and automatic mappings you have done so far).
- Please explain the data curation workflow better, clearly specifying the different steps before moving to a description of each. CLDF is presented in different places and I am still not sure I understand how it is used for the present datasets -- again, examples would help. Also please distinguish clearly in the text between the format and the functionalities it affords.
- CLDF, CSVW, CLLD, ... it gets confusing, please help the reader.
- Please also clarify why you release datasets in two formats (CLDF, another -- which?).
- Also please include a brief note on how CLDF relates to other standardization efforts such as TEI.

ASPECT TO IMPROVE #2: LESS TECHNICAL DETAIL

- Please remove technical details that belong in the documentation, not the article. Two examples:
  - 1) In the description of the Python packages, details such as "Typing *concepticon -h* will give a list of the functionalities." Important: Make sure that the information that is relevant to understand the functionalities of the packages remains.
  - 2) You include links to many specific files in the repositories. In the article, you should provide only the main links and the information that is relevant to understand what they contain. For where to find which specific kind of information, you should rely on the documentation in the repositories.
- Are you sure Table 1 is needed? If so, expand the information you provide -- e.g. line 2 is not understandable ("Concept lists that contain further annotations which exceed the complexity of ranks"?).

ASPECT TO IMPROVE #3: STRUCTURE

 The way the information is presented in the current version makes it difficult for the reader to understand the material. I list here what struck me the most, but the article needs to be thoroughly revised in this respect.

- The abstract mixes specific considerations about the historical development of the dataset that is being presented with general motivation and with relevant aspects of the dataset. The line "The Concepticon is based..." talks about the Concepticon as if it were already familiar to the reader.
- In the introduction, after a 3-sentence general motivation, the article jumps into details without giving the general picture: "For this reason, a community-led initiative developed the Cross-Linguistic Data Formats (CLDF, Forkel et al., 2018) [...] The CLDF format specifically targets interoperability and reusability..."
- "test-driven" is mentioned in the abstract and the keywords, but then it's not introduced explicitly in the article: the first mention is "This is the advantage of a test-driven data curation workflow" -- please explain what a test-driven data curation workflow is beforehand.
- Please revise the structuring into sections: e.g., should section "Consistency and transparency through CLDF" really at the same level as Intro, Materials and Methods, etc.? Can you use a more transparent title? (This section is particularly unclear.) How about the subsections?
- More generally, please remove the many redundancies in the text (including two long links to the markdown file containing the list of contributors!).

CONCLUSION

 While the work has merit, I cannot approve the article in its present form. I am looking forward to a revised version.

 ---

 Typos/other:

- Figure 1 really helps! the "arbitrarité" part was confusing until I understood it was the logo for Concepticon. It's not the most transparent logo. :) (By the way, on the https://concepticon.clld.org/ website it says "arbitraire".)
- The text in Figure 2 is too small to be read.
- p. 3, "preface" ==> "surface".
- p. 4, "The different contents of the lists", not clear what "the lists" refers to here.
- p. 5, "These correlation studies not only show interesting patterns arising from the data such as similarities in word frequencies across diverse languages (Tjuka, 2020b) but also illustrate that cross-linguistic comparable datasets for particular word properties such as sensory modality are not available yet" ==> ?
- p. 7, "NoRaRe also allows the mapping of typed data such as networks, which is an added benefit compared to the data in Concepticon": what do you mean with "typed data such as networks"? Allows, or has been done already?
- p. 7, "the fact that CLDF datasets can be converted to relation databases turning relations between tables into foreign key constraints implies that valid CLDF datasets will have unique identifiers for each row of a table": please rephrase in non-technical terms and explain why this is good.
- p. 7, is "careless" really what you mean here?

Are sufficient details of methods and materials provided to allow replication by others?

No

Is the rationale for creating the dataset(s) clearly described?

Yes

Are the datasets clearly presented in a useable and accessible format?

Yes

Are the protocols appropriate and is the work technically sound?

Partly

Reviewer Expertise:

Semantics, lexicon, linguistics, computational linguistics, cognitive science

I confirm that I have read this submission and believe that I have an appropriate level of expertise to state that I do not consider it to be of an acceptable scientific standard, for reasons outlined above.

# Response

**Published:** 2023-02-21

**Authors:** Annika Tjuka

**Affiliations:** Department of Linguistic and Cultural Evolution, Max Planck Institute for Evolutionary Anthropology, Leipzig, Germany

We would like to thank the reviewer for the thorough assessment of our manuscript. We will respond in detail to all points raised and prepare a new version as soon as all reviews are available.

# Response

**Published:** 2023-04-21

**Authors:** Annika Tjuka

**Affiliations:** Department of Linguistic and Cultural Evolution, Max Planck Institute for Evolutionary Anthropology, Leipzig, Germany

We would like to thank the reviewer for the detailed comments on our manuscript. We have provided more information in the revision and modified the structure of the manuscript. However, we specifically choose the category “Data Note”  to provide technical details on the data curation workflow and the relevant Python packages. We, therefore, kept the relevant passages in the new version of the manuscript. We address the reviewers' comments in detail below (order: reviewer comment, our response, implemented change) so that our decisions are transparent, and hope that they are okay with us not following all the suggestions. We try to take into account the specific article format specified by the journal, which we believe is partly in conflict with some of the suggestions in the review.  

- Please include a table with relevant descriptive statistics of the datasets in their 3.0 and 1.0 releases (minimally n. of datasets, concepts, words, languages, language families).

We thank the reviewer for the suggestion. In addition to the descriptive statistics mentioned in the text, we added a table (Table 3) so that the data can be conveniently compared.  

- The main notions around which the data are organized should be more clearly explained, with examples. The notion of "concept set" was particularly hard for me to understand; I had to check the websites, and even there it took some digging: The example on the homepage of the CLLD web app ( https://concepticon.clld.org/) talks about the concept set SIBLING, so it looks like the other concepts in the network shown (OLDER SIBLING, YOUNGER BROTHER, ...) are members of this set. However, if one queries the interface, OLDER SIBLING and YOUNGER BROTHER are themselves concept sets. It turns out that "concept set" does not refer to sets of concepts in Concepticon, but to sets of concepts defined in the primary datasets. Please explain and illustrate how you use the following terms: "concept", "concept set", "concept label", "concept list". Also explain how these terms relate to words as commonly defined by linguists, and what the place of words is in the Concepticon (including improving the claim that "concept lists look like a list of words. However, words represent concepts in the mind" -- check e.g. G. Murphy's chapter on word meaning in The Big Book of Concepts).

The reviewer is correct, and we noticed that the terminology used in linguistics does not always match the terminology used in psychology. Therefore, some translation work is required. To avoid confusion about the terminology and abbreviations used in our manuscript, we have included a glossary (Table 1) of relevant terms and their definitions.

  

- Also provide information about the ontological categories: how was the set of categories defined, and how were they assigned to specific concepts?

The ontological categories are based on the categories used by the World Loanword Database (Haspelmath and Tadmor 2009). However, they were modified regarding the concrete terms used, in order to make clear that we do not talk about concrete parts of speech since we are dealing with concepts and not with words (see List et al. 2016: 2394). 

  

- The mapping between the primary datasets and Concepticon includes an automatic step: "the algorithm checks the elicitation glosses that are mapped to a given concept set in Concepticon and compares them with the word in a given list. (...) If there is a match, the word is mapped." Please explain how the checking and comparing is made, and what the criteria are to decide whether there is a match, at an adequate level of abstraction for the present report. Also, explain "elicitation glosses" -- I thought they were definitions but Figure 1 suggests they are words. Maybe it depends on the dataset?

We added a definition of “elicitation gloss” in the glossary (Table 1) at the beginning of the manuscript. We hope that the definition makes it clear that the automatic check compares word forms. The examples in the sentence should make the comparison more transparent.

*"For this procedure, the algorithm checks the elicitation glosses that are mapped to a given concept set in Concepticon (e.g., tree, árbol) and compares them with the word in a given list (e.g., tree, forest, wood). "*  

- Also please provide information on how many datapoints from the primary sources are left unlinked at present (after the manual and automatic mappings you have done so far).

We added an example illustrating our decision to unlink a given concept. However, we would like to refrain from stating the number of unlinked data points. As the Concepticon and NoRaRe are constantly being developed, we add new links in each version and also update the links, for example, when a new concept set is introduced. We update our summary statistics with each release and the most recent numbers are provided here: https://github.com/concepticon/concepticon-data/tree/v3.0.0/concepticondata  Furthermore, data points which are not linked can also be inspected on our web page directly: when looking up the concept set “0 ”, one can see that we have 202 concept lists in which elicitation glosses are not linked to concept sets. These amount to more than 20,000 elicitation glosses ( https://concepticon.clld.org/parameters/0). Given that Concepticon by now comprises 120,000 elicitation glosses in total, we succeed in linking more than 80% of the data.

*"For example, if a list contains the elicitation gloss smoke without any further information about whether the verb or noun meaning is meant, we do not map it to one of the concept sets 778 SMOKE (EXHAUST) or 1689 SMOKE (INHALE). Elicitation glosses which are not assigned to a concept set are automatically linked to the concept set 0 NA."*  

- Please explain the data curation workflow better, clearly specifying the different steps before moving to a description of each. CLDF is presented in different places and I am still not sure I understand how it is used for the present datasets -- again, examples would help. Also please distinguish clearly in the text between the format and the functionalities it affords.

We recognize the reviewer's concern about the structure and presentation of our descriptions. We, therefore, moved the description of the Python packages pyconcepticon and pynorare below “Manual and automated mapping” in the “Materials and Methods” section. In this way, the information about the CLDF format is not interrupted by the description of functionalities. Due to the scope of the Data Note, we cannot provide an extensive description of the workflows but we added a note at the beginning of the section “Manual and automated mapping” where interested readers can find more details. Furthermore, we added references to our blog posts in which we provide tutorials on how to use the Python packages (see also comments below and the comment by Reviewer 2).  

*"Detailed descriptions of our workflows are provided in the articles introducing Concepticon (List et al., 2016b) and NoRaRe (Tjuka et al., 2022a) as well as in the tutorials on our blog (Tresoldi, 2019a, Tresoldi, 2019b; Tjuka, 2020a). Here, we summarize the basic steps of the manual and automated mapping workflows."* *"Typing concepticon -h will give a list of the functionalities, for example, create_metadata which automatically creates the metadata JSON file. Guides on how to use the functionalities can be found on our blog (Tresoldi, 2019a, Tresoldi, 2019b; Tjuka, 2020a)."*  

- CLDF, CSVW, CLLD, ... it gets confusing, please help the reader.

We added all abbreviations in the glossary (Table 1) at the beginning of the manuscript.

  

- Please also clarify why you release datasets in two formats (CLDF, another -- which?).

We clarified the reasons for releasing CLDF versions of the data in the first paragraph of the section “CLDF datasets”.  

*"With the major release of Concepticon Version 3.0 (List et al., 2022a) and NoRaRe Version 1.0 (Tjuka et al., 2022b), both resources were also made available as CLDF datasets (List et al., 2022b; Tjuka et al., 2022c). While the raw data is updated and improved consistently in the respective GitHub repositories, the CLDF datasets allow for the reuse and exploration of the data in a different way. The data in the CLDF datasets are represented in tabular format and the corresponding metadata in JSON format. The bundling of the data makes it possible to represent relations between the tables. The data model is available here: github.com/concepticon/concepticon-cldf/blob/v3.0.0/cldf/README.md. By releasing the data in Concepticon and NoRaRe as CLDF datasets,  tools such as csvkit (pypi.org/project/csvkit/1.1.1) can be used to process and analyse the data conveniently. Examples of how to use the Concepticon data from the CLDF dataset can be found under github.com/concepticon/concepticon-cldf/tree/v3.0.0/doc and for NoRaRe under github.com/concepticon/norare-cldf/tree/v1.0.0."*

  

- Also please include a brief note on how CLDF relates to other standardization efforts such as TEI.

We added a footnote in which we refer readers interested in the major ideas behind CLDF and how they contrast with TEI to our original publication introducing CLDF from 2018 (Forkel et al. 2018). *Footnote: "We refer readers interested in the major design principles behind CLDF and the contrasts with other standardization efforts to the original publication introducing CLDF by Forkel et al. (2018)."*  

- Please remove technical details that belong in the documentation, not the article. 

We thank the reviewer for the suggestion but we decided to keep relevant technical details in the manuscript. The reason for the decision is that we consciously choose the category “Data Note” instead of “Research Article” so that we could provide a text that includes all necessary information in one place. While we also provide documentations on GitHub, we noticed that researchers tend to rely on published papers. Further details on how to use specific functionalities can also be found on our blog Computer-Assisted Language Comparison in Practice ( https://calc.hypotheses.org/). The data note was intended to bring these different resources together and provide a reference point for researchers who are interested in using our resources. The links to the documentation in the GitHub repository are as follows:

1. General overview: https://github.com/concepticon/concepticon-data/blob/master/README.md
2. Getting started document including tutorials on the blog Computer-Assisted Language Comparison in Practice: https://github.com/concepticon/concepticon-data/blob/master/GettingStarted.md 
3. Contributing procedure: https://github.com/concepticon/concepticon-data/blob/master/CONTRIBUTING.md 
4. Release procedure: 
  - https://github.com/concepticon/concepticon-data/blob/master/RELEASING.md 
  - https://github.com/concepticon/norare-data/blob/master/RELEASING.md 

- Two examples: 1) In the description of the Python packages, details such as "Typing concepticon -h will give a list of the functionalities." Important: Make sure that the information that is relevant to understand the functionalities of the packages remains.

We added an example of a functionality and further references to tutorials on our blog.  

*Typing concepticon -h will give a list of the functionalities, for example, create_metadata which automatically creates the metadata JSON file. Guides on how to use the functionalities can be found on our blog (Tresoldi, 2019a, Tresoldi, 2019b; Tjuka, 2020a)."*  

- 2) You include links to many specific files in the repositories. In the article, you should provide only the main links and the information that is relevant to understand what they contain. For where to find which specific kind of information, you should rely on the documentation in the repositories.

We thank the reviewer for the suggestion but we decided to keep the links in the manuscript. As mentioned in the comment above, the Data Note is intended to provide a reference point so that researchers find all relevant information in one place. Linking to the various files in the GitHub repository and documentation is a way for us to bundle the information that already exists. We hope that researchers can use the Data Note as an entry point to explore our resources in more detail.

  

- Are you sure Table 1 is needed? If so, expand the information you provide -- e.g. line 2 is not understandable ("Concept lists that contain further annotations which exceed the complexity of ranks"?).

We included Table 1 (now Table 2) because the tags have been updated since the last publication in List (2018) and we wanted to incorporate the new tags to make it easier for researchers to find the data they are looking for. In addition, the table provides descriptive statistics and examples of lists available in Concepticon and NoRaRe. However, we recognise that some of the descriptions were not sufficient and improved them accordingly.

  

- The way the information is presented in the current version makes it difficult for the reader to understand the material. I list here what struck me the most, but the article needs to be thoroughly revised in this respect. The abstract mixes specific considerations about the historical development of the dataset that is being presented with general motivation and with relevant aspects of the dataset. The line "The Concepticon is based..." talks about the Concepticon as if it were already familiar to the reader.

We revised the abstract according to the reviewer’s suggestion.  

*Abstract: "Language comparison requires user-friendly tools that facilitate the standardization of linguistic data. We present two resources built on the basis of a standardized cross-linguistic format and show how the data is curated and extended. The first resource, the Concepticon, is a reference catalog for standardized concepts from linguistic research. While curating the Concepticon, we found that a variety of studies in distinct research fields collected information on word properties. However, until recently, no resource existed that contained these data to enable the comparison of the different word properties across languages. This gap was filled by the Database of Norms, Ratings, and Relations (NoRaRe), which is an extension of the Concepticon. Here, we present the major release of both resources - Concepticon Version 3.0 and NoRaRe Version 1.0 - which represents an important step in our data development. We show that extending and adapting the data curation workflow in Concepticon to NoRaRe is useful for the standardization of cross-linguistic datasets. In addition, combining datasets from different research fields enables studies grounded in language comparison. Concepticon and NoRaRe include lexical data for various languages, tools for test-driven data curation, and the possibility for data reuse. The first major release of NoRaRe is also accompanied by a new web application that allows convenient access to the data."*

  

- In the introduction, after a 3-sentence general motivation, the article jumps into details without giving the general picture: "For this reason, a community-led initiative developed the Cross-Linguistic Data Formats (CLDF, Forkel et al., 2018) [...] The CLDF format specifically targets interoperability and reusability..."

We included a sentence which facilitates the transition between the two sentences. *"Therefore, it is necessary to create a standardized format that applies to all languages, and at the same time, to provide the tools for effortless standardization. A community-led initiative has recognized this need and developed the Cross-Linguistic Data Formats (CLDF, Forkel et al., 2018)."*

  

- "test-driven" is mentioned in the abstract and the keywords, but then it's not introduced explicitly in the article: the first mention is "This is the advantage of a test-driven data curation workflow" -- please explain what a test-driven data curation workflow is beforehand.

We added a sentence in the introduction to clarify what “test-driven” entails. *"We apply a test-driven data approach, i.e., specific tests are carried out that match the formal requirements of the data automatically with the specifications developed in CLDF."*  

- Please revise the structuring into sections: e.g., should section "Consistency and transparency through CLDF" really at the same level as Intro, Materials and Methods, etc.? Can you use a more transparent title? (This section is particularly unclear.) How about the subsections?

We revised the structuring into sections according to the reviewer’s comments above so that the descriptions of the Python packages are in the “Materials and methods” section. We used the section on CLDF to integrate the subsections on “Using and representing the data” under one umbrella. We also updated the heading of the section. *Heading: "Increasing reusability through Cross-Linguistic Data Formats"*

  

- More generally, please remove the many redundancies in the text (including two long links to the markdown file containing the list of contributors!).

We thank the reviewer for this comment, which emphasizes the relevance of readability. Due to the style guide of the Open Research Europe Journal, we have included all links in the text rather than in footnotes. Since we cannot assume that everyone will read the text from beginning to end, we decided to add the links in multiple places so that the data can be conveniently accessed. We also considered deleting the link to the contributors but decided to keep it. The data collection in Concepticon and NoRaRe is a collaborative effort and the link to contributors highlights how many people have contributed over the years. 

  

- While the work has merit, I cannot approve the article in its present form. I am looking forward to a revised version.

We thank the reviewers for their valuable comments which contributed to improving the manuscript. We addressed all comments and uploaded a revised version of the manuscript.  

- Figure 1 really helps! the "arbitrarité" part was confusing until I understood it was the logo for Concepticon. It's not the most transparent logo. :) (By the way, on the https://concepticon.clld.org/ website it says "arbitraire".)

We exchanged the logo in the figure with the logo from the Concepticon website.

  

- The text in Figure 2 is too small to be read.

We enlargened the part of the figure which was relevant to illustrate the difference on the web page.

  

- p. 3, "preface" ==> "surface".

We changed the typo. *"On the surface, concept lists look like a list of words." *  

- p. 4, "The different contents of the lists", not clear what "the lists" refers to here.

We added further information to specify which lists were meant. *"The different contents of the concept lists in Concepticon are accounted for by giving them tags which make it more straightforward to search for a particular kind of data (List, 2018)."*  

- p. 5, "These correlation studies not only show interesting patterns arising from the data such as similarities in word frequencies across diverse languages (Tjuka, 2020b) but also illustrate that cross-linguistic comparable datasets for particular word properties such as sensory modality are not available yet" ==> ?

We paraphrased the sentence. *"For example, we compared word frequencies across English, German, and Chinese, and the results showed that word frequencies were similar (Tjuka, 2020b). Another study compared sensory modality across English, Dutch, and Italian showing subtle differences in each sensory modality (Tjuka, 2022). Thus, the database can be used to compare word properties and reveal both cross-linguistic similarities and differences."*

  

- p. 7, "NoRaRe also allows the mapping of typed data such as networks, which is an added benefit compared to the data in Concepticon": what do you mean with "typed data such as networks"? Allows, or has been done already?

We specified the data type. *"NoRaRe also includes mappings of typed data, for example, entries in Wikidata (www.wikidata.org) and BabelNet (www.babelnet.org), which is an added benefit compared to the data in Concepticon."*  

- p. 7, "the fact that CLDF datasets can be converted to relation databases turning relations between tables into foreign key constraints implies that valid CLDF datasets will have unique identifiers for each row of a table": please rephrase in non-technical terms and explain why this is good.

We rephrased the sentence with less technical terminology. *"CLDF datasets follow the data model of relational databases, where tables can link to each other using foreign keys. This means that CLDF datasets can automatically be converted to relational databases to facilitate efficient data access. It also means that CLDF datasets follow the recommendations for “tidy” data (Wickham 2014; Wilson et al., 2017), in particular by providing unique identifiers for each row of a table."*  

- p. 7, is "careless" really what you mean here?

We deleted the adjective from the sentence. *"Especially, if a new concept list or improvements on existing data are added, the tests that run with the command concepticon test can spot inconsistent mappings, missing files, incorrect numbering of the concept sets, and many more mistakes."*
